# Phytochemical Analysis, Antioxidant and Antibacterial Activities, Minerals Element Profiling, and Identification of Bioactive Compounds by UPLC-HRMS Orbitrap in Four Aromatic and Medicinal Plants

**DOI:** 10.3390/molecules30061279

**Published:** 2025-03-12

**Authors:** Aicha Boubker, Abdelmoula El Ouardi, Taha El Kamli, Adnane El Hamidi, Mohammed Kaicer, Faouzi Kichou, Najia Ameur, Khaoula Errafii, Rachid Ben Aakame, Aicha Sifou

**Affiliations:** 1 Laboratory of Materials, Nanotechnologies and Environment, Faculty of Sciences, Mohammed V University in Rabat, BP: 1014, Ibn Battouta Avenue, Rabat 10000, Morocco; aicha.boubker@um5r.ac.ma (A.B.); a.elhamidi@um5r.ac.ma (A.E.H.); 2 Department of Microbiology of Water, Food and Environment, National Institute of Hygiene, BP: 769, Agdal, 27, Avenue Ibn Batouta, Rabat 10000, Morocco; abdoelouardi@yahoo.fr (A.E.O.); najiaameur22@gmail.com (N.A.); 3 Laboratory of Anti-Doping Control, Hassan II Institute of Agronomic and Veterinary, BP: 6202, Madinat Al Irfane, Rabat 10000, Morocco; elkamlit@yahoo.fr; 4 Laboratory of Analysis, Geometry and Applications, Systems and Optimization, Ibn Tofail University, Campus of University, BP: 242, Kenitra 14000, Morocco; mohammed.kaicer@uit.ac.ma; 5 Department of Pathology and Veterinary Public Health, Hassan II Institute of Agronomic and Veterinary, BP: 6202, Madinat Al Irfane, Rabat 10000, Morocco; kichou.faouzi@gmail.com; 6 African Genom, University Mohammed VI Polytechnic Center, BP: 660, Hay Moulay Rachid, Benguerir 43150, Morocco; khaoula.errafii@um6p.ma; 7 Laboratory of Food Toxicology, National Institute of Hygiene, BP: 769, Agdal, 27, Avenue Ibn Batouta, Rabat 10000, Morocco; benakame@yahoo.fr

**Keywords:** antimicrobial activity, antioxidant activity, bioactive compounds, GF-SAA, phytochemical analysis, traces elements, UPLC–HRMS orbitrap

## Abstract

Four aromatic and therapeutic plants, *Thymus vulgaris*, *Rosmarinus officinalis*, *Pimpinella anisum*, and *Foeniculum vulgare*, were examined in this comparative study. The objectives were to assess its phytochemical composition; polyphenol, flavonoid, and tannin content; antioxidant and antibacterial activity; bioactive molecule identification; and critical trace element quantification. Its aqueous and organic extracts were examined, focusing on ethanolic extracts. The ethanolic extract’s ability to neutralize free radicals was validated by phytochemical studies and antioxidant tests, underscoring their role in preventing oxidative stress. An Ultra-Performance Liquid Chromatography—High-Resolution Mass Spectrometry Orbitrap Exploris 120 (UPLC–HRMS Orbitrap) was used to identify the bioactive chemicals, and the results showed a variety of compounds having antibacterial and antioxidant properties. The important trace elements found in these plants were also measured using a Graphite Furnace-Atomic Absorption Spectrometer (GF-AAS). These components are essential to the biological characteristics of the plants, especially their antioxidant and antibacterial capacities. Among the aqueous extracts, it was observed that *Rosmarinus officinalis* and *Foeniculum vulgare* exhibited a MIC of 3.91 µg/mL against *Staphylococcus*. Additionally, *R. officinalis* also demonstrated a MIC of 3.91 µg/mL against *Escherichia coli*. All of the data were interpreted and shown using principal component analysis. The results were grouped and explained using this statistical method, which revealed a strong association between the abundance of antibacterial and antioxidant chemicals in the four plants under investigation.

## 1. Introduction

Traditional medicinal and aromatic herbs have been used for therapeutic purposes for centuries. In recent decades, interest in these natural remedies has resurged due to their potential health benefits and bioactive properties [1]. Morocco’s medicinal and aromatic plants (MAPs) are vital for traditional medicine and rural economies. However, rising global demand and overexploitation threaten their sustainability. Urgent conservation and sustainable management strategies are needed [2]. The loss of traditional knowledge regarding their harvesting and preparation methods further threatens their conservation and long-term availability [3].

A total of 743 species, classified into 101 families and 371 genera, have been documented and are used in traditional Moroccan phytotherapy. The families most represented are Asteraceae (10.92%), *Lamiaceae* (10.78%), *Fabaceae* (5.93%), and *Apiaceae* (5.12%). Native (AMPs) in Morocco account for 40 taxa or 5.39% of the total taxa [4]. The *Thymus vulgaris* L. and *Rosmarinus officinalis* L. belong to the *Lamiaceae* family, which is widespread and one of the most abundant among flowering plants. This family is well-known for its many species with medicinal properties [5]. These characteristics have attracted growing interest in the veterinary industry, especially in reducing antibiotic usage and enhancing agricultural animals’ zootechnical performance [6].

*T. vulgaris* essential oil is a prospective medicinal agent since it is high in thymol and carvacrol and has potent anti-inflammatory and antibacterial qualities by blocking pro-inflammatory cytokines and rupturing bacterial membranes [7]. Ethnobotanical and pharmacological studies suggest that the bioactive compounds of medicinal plants, such as *R. officinalis*, could contribute to the development of novel plant-based therapies in modern medicine and have been identified for their potential anticancer properties [8]. *R. officinalis* and *F. vulgare* are used as medicinal plants for managing type 2 diabetes in Moroccan phytotherapy, mainly as infusions for metabolic regulation [9]. The essential oils of *F. vulgare* and *P. anisum*, rich in bioactive chemicals, serve as antimicrobial agents and digestive stimulants. These oils not only support animal growth and weight gain but also serve as a natural alternative to antibiotics in feed, significantly enhancing daily weight gain and overall body weight [10,11].

In this study, we compared four medicinal plant species using several analytical criteria, including a phytochemical assay, antioxidant capacity, identification of bioactive molecules, essential minerals, and antibacterial efficacy against common microorganisms. This study provides novel insights into the bioactive potential of these crude plant extracts by evaluating their antioxidant and antimicrobial activities. Using UPLC–HRMS, we identify key bioactive compounds, highlighting their potential as natural alternatives to antibiotics. This comparison provides an in-depth understanding of the pharmacological properties and therapeutic potential of the species studied and paves the way for future research into their use in phytotherapy. To evaluate the relationships between these parameters and identify the characteristics that distinguish the species, we employed a principal component analysis (PCA). This multivariate statistical approach allowed us to effectively highlight the diversity of plant characteristics, their commonalities, and their unique features. A thorough understanding of their pharmacological properties and therapeutic potential thus opens the door to future studies on their use in phytotherapy.

## 2. Results and Discussions

### 2.1. Total TPC, TFC and TCT

Based on the phytochemical results obtained in Table 1, it was found that the highest concentrations of TPC, TFC, and TCT in the four plants studied were associated with extracts obtained with ethanol. This finding suggests that ethanol is particularly effective as an extraction solvent for these bioactive compounds. These findings are consistent with the fact that ethanol promotes better the extraction of polyphenols and flavonoids [12,13,14]. The TPC of *T. vulgaris* in different extracts is higher than that reported in a study on different Thymus species, where *T. vulgaris* had a TPC of 35.73 μg GAE/mg dry weight [15]. The TFC and TPC of the ethanolic extract of *R. officinalis* in our study were found to be 208.46 ± 0.16 μg QE/mg and 75.79 ± 0.02 μg GAE/mg, respectively. These values are significantly higher than those reported in a previous study [16], where the ethanolic extract contained 72.88 ± 3.84 mg QE/g and 52.50 ± 2.75 mg GAE/g. Additionally, the ethanolic extract of *P. anisum* in our study (67.71 ± 0.04 μg GAE/mg E) exhibited a significantly higher TPC than the 7.5 ± 0.16 mg GAE/g reported by [14]. Furthermore, ethanol extraction of *F. vulgare* seeds resulted in higher concentrations of TPC and TFC compared to water extraction, as reported in the study [17]. Ethanol’s solubility and polarity properties allow for the extraction of a wide range of phytoconstituents, making it more effective than other solvents.

### 2.2. Antioxidant Activity

Additionally, as shown in Table 2, the ethanol extracts exhibited a lower Concentration of Inhibition 50% (CI50) value compared to the other extracts. This indicates a greater ability of ethanolic extracts to neutralize free radicals, which is a key indicator of their antioxidant potential. According to the article [18], ethanol extraction of *T. vulgaris* provides better antioxidant activity compared to other solvents. The IC_50_ value obtained from extracts of *R. officinalis* is significantly lower than the values reported in reference [19], which range from 95.32 to 172.80 µg/mL. Moreover, ascorbic acid, used as the reference substance and diluted in ethanol, exhibited an IC_50_ value of 4.12 µg/mL. These results reinforce that ethanol is effective not only in extracting phytochemicals but also in preserving their antioxidant properties.

### 2.3. Determination of Bioactive Molecules by UPLC–HRMS Orbitrap

Table 3 presents various chemical compounds, each with distinct bioactivities. This allows us to explore their therapeutic potential and applications in medicine and nutrition. The results show that several of these compounds possess significant antibacterial and antioxidant properties. These properties are crucial for the development of new therapies and dietary supplements. Oleanolic acid was identified in the four analyzed plants, confirming its widespread presence in the plant world. This pentacyclic triterpene is widely recognized for its multiple pharmacological properties, including hepatoprotective, anti-inflammatory, antioxidant, and anticancer effects [20]. Thymol, ursolic acid, carvone, trans-anethole, apigenin, and caffeic acid are known for their potent antioxidant and antimicrobial properties, playing a key role in protecting against oxidative stress and combating various pathogens [21]. These natural bioactive compounds have various therapeutic applications and can be used in the creation of dietary and pharmaceutical products [22]. Additionally, they give rise to substances like terpenes and flavonoids, offering innovative and eco-friendly solutions for the pharmaceutical and nutraceutical sectors [23]—furthermore, advancements in extraction methods and potential avenues for the sustainable use of marine resources in functional foods, cosmetics, and medicine [24].

### 2.4. Mineral Contents in the Plants

Table 4 presents the concentrations of macroelements and trace elements in four plants: *T. vulgaris*, *R. officinalis*, *P. anisum*, and *F. vulgare*. Analysis of the data reveals significant differences in the mineral profiles of each plant, which may have implications for their nutritional and therapeutic properties. *T. vulgaris* contains relatively high concentrations of Ca (20.823 ± 0.71 mg/g) and Fe (0.97 ± 0.11 mg/g), which are significantly higher compared to the other plants. These variations could be explained by environmental and methodological factors [21]. However, *P. anisum* shows a slightly higher Na content, as observed in other studies [22]. This may be attributed to the impact of climate change, particularly through increased salinity [23]. In contrast, the other plants exhibited much lower Fe concentrations. *F. vulgare* stood out with a remarkably high K concentration (36.413 ± 0.98 mg/g), compared to the other plants. Additionally, *F. vulgare* contained the highest Zn concentration (0.041 ± 0.005 mg/g). These results confirm that this plant is particularly rich in K and Zn [24].

### 2.5. Antibacterial Activity

Bacteria play a crucial role in both clinical and dietary contexts, with some species being particularly significant due to their pathogenicity and prevalence. Among the bacteria we have selected for this study are *E. coli*, *Salmonella*, and *Staphylococcus*. *E. coli* is known for its propensity to develop resistance and its frequent involvement in intestinal and urinary tract infections, making it a model bacterium for testing antibacterial agents [48]; *Salmonella*, a major cause of foodborne diseases, is commonly used to assess the antibacterial activity of plant extracts [49]. *Staphylococcus* is often linked to skin, respiratory, and foodborne illnesses [50]. The addition of *T. vulgaris*, *R. officinalis*, and *F. vulgare* essential oils significantly improved the total aerobic mesophilic flora, fecal and total coliforms, as well as the digestive microbiota [51]. Ciprofloxacin produced an average inhibition zone of 28.00 ± 2.00 mm for *E. coli*, Gentamicin showed 23.00 ± 1.00 mm against *Salmonella*, and Oxacillin produced 24.00 ± 1.00 mm against *Staphylococcus*.

Table 5 presents the results, which demonstrate significant variations in the antimicrobial efficacy depending on the plant, concentration, and type of bacteria. *F. vulgare* exhibited the largest zones of inhibition, particularly against *Staphylococcus* (15.50 mm at 100 mg/mL) and *E. coli* (14.50 mm at 100 mg/mL). This suggests that F. vulgare has exceptional antimicrobial activity, with higher efficacy than the other extracts tested. In comparison, *P. anisum* shows relatively low antimicrobial activity, with low or no inhibition zones across all concentrations tested. Extracts of *T. vulgaris* and *R. officinalis* showed an increase in inhibition zones as the concentration increased, although this effect was not consistent for all bacteria. At lower concentrations, the antimicrobial activity decreased, highlighting that the effectiveness of the extracts depends on their concentration [52]. *E. coli* and *Staphylococcus* were generally more sensitive to the plant extracts than *Salmonella* [53] with wider zones of inhibition observed against these two strains. This suggests that *Salmonella* may be more resistant to the antimicrobial compounds present in these extracts. It is noteworthy that the distilled water extracts showed no antimicrobial activity at any concentrations tested, confirming that the observed zones of inhibition were not due to contaminants or non-specific effects but rather to the active compounds present in the plant extracts. Regarding the MIC values, the analysis was conducted in triplicate. Ciprofloxacin had a MIC of 0.015 to 0.03 µg/mL for *E. coli*, Gentamicin ranged 5 to 1 µg/mL for *Salmonella*, and Oxacillin had a MIC of 0.25 to 2 µg/mL for *Staphylococcus*.

Table 6 presents the MIC values for the four extracts, indicating the minimum concentration required to inhibit bacterial growth and providing an overview of the antimicrobial efficacy of the extracts tested. *R. officinalis* stood out for its low MIC values, particularly against *E. coli* and *Staphylococcus*, with an MIC of 3.91 µg/mL. This suggests that *R. officinalis* has significantly higher antimicrobial activity compared to the other extracts tested. *T. vulgaris* also showed good efficacy, with an MIC of 7.81 µg/mL against *E. coli*, 31.25 µg/mL against *Salmonella*, and 15.62 µg/mL against *Staphylococcus. P. anisum* exhibited very high MIC values (125 µg/mL) for all the bacterial strains tested, indicating relatively low antimicrobial efficacy. *Staphylococcus* appeared to be more sensitive to *F. vulgare* and *R. officinalis* extracts, with MIC values as low as 3.91 µg/mL, while *Salmonella* required higher concentrations for effective inhibition, particularly for *T. vulgaris* and *F. vulgare* extracts. These results suggest that the antibacterial activity of essential oils from these plants is effective against a variety of harmful bacteria [50]. The synergistic potential of plant extracts in boosting antibacterial activity has been extensively documented in various studies [54].

### 2.6. Correlation Matrix

**Figure 1:**
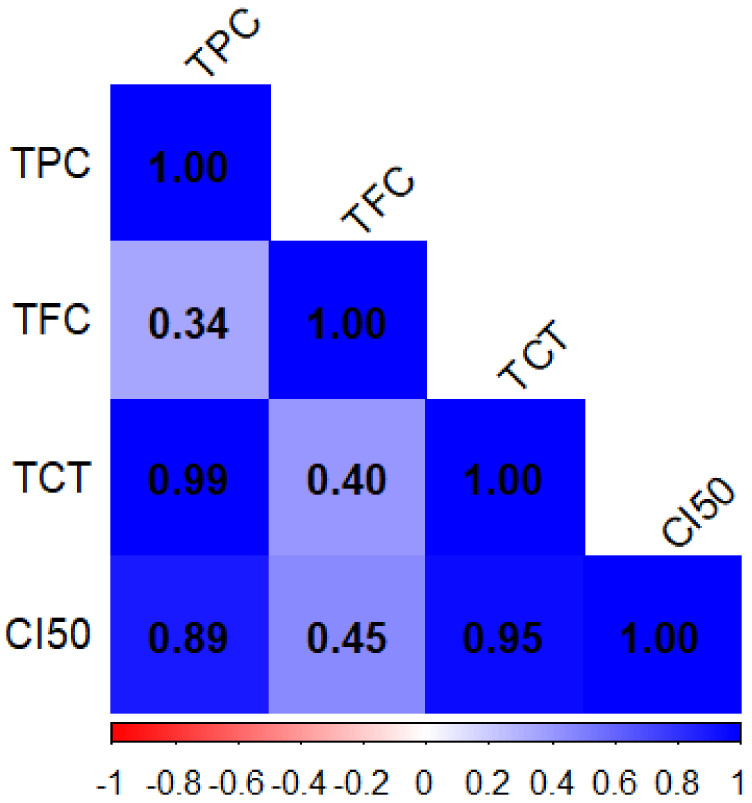
The coefficients of the Pearson correlation matrix are between the variables: phenolic compound content (TPC, TFC, and TTC) and antioxidant activity of *T. vulgaris*, *R. officinalis*, *P. anisum*, and *F. vulgare*.

**Figure 2:**
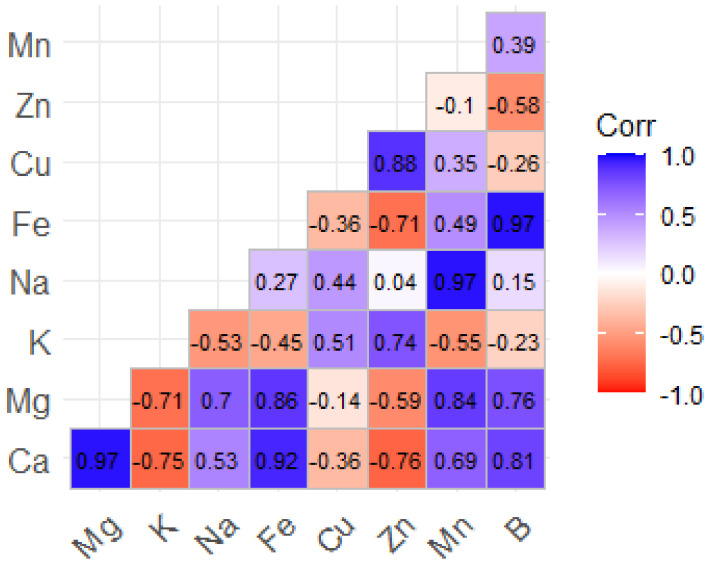
Coefficients of the correlation matrix between macroelements (Ca, Mg, K, and Na) and trace elements (Fe, Cu, Zn, Mn, and B) in *T. vulgaris*, *R. officinalis*, *P. anisum*, and *F. vulgaire*.

Figure 1 presents the Pearson correlation coefficients for phenolic compound contents (TPC, TFC, and TCT) and antioxidant activity. Similarly, Figure 2 displays the Pearson correlation coefficients for macroelements (Ca, Mg, K, and Na) and trace elements (Fe, Cu, Zn, Mn, and B). Significant correlations between the various variables under study are displayed in the correlation matrix. TPC and TCT (r^2^ = 0.99) suggest that these two metrics change in nearly the same way. Furthermore, a strong association was found between IC_50_, a measure of antioxidant activity (after inversion), and both TCT (r^2^ = 0.95) and TPC (r^2^ = 0.89), highlighting the significant impact of these compounds on antioxidant activity. In contrast, the moderate correlation (r^2^ = 0.45) between TFC and CI_50_ indicates that flavonoids may contribute to enhancing antioxidant activity. Lastly, a weak correlation (r^2^ = 0.34) between TPC and TFC was found, suggesting that these two chemical categories differ from one another. Thus, the findings highlight the significance of TPC and TCT as key factors influencing antioxidative efficacy.

The second matrix in Figure 2 highlights the strong positive correlations between Ca and Mg (r^2^ = 0.97) and between Na and Fe (r^2^ = 0.97). However, there are notable negative correlations between Ca and K (r^2^ = −0.75) and Mg and K (r^2^ = −0.71), suggesting that there may be competition for uptake. Fe and Zn have an adverse association (r^2^ = −0.71), whereas Zn and Cu have a substantial correlation (r^2^ = 0.88), indicating a synergistic interaction. These findings demonstrate strong correlations between polyphenols, antioxidant capacity, and metal-to-metal interactions.

### 2.7. Principal Component Analysis

**Figure 3:**
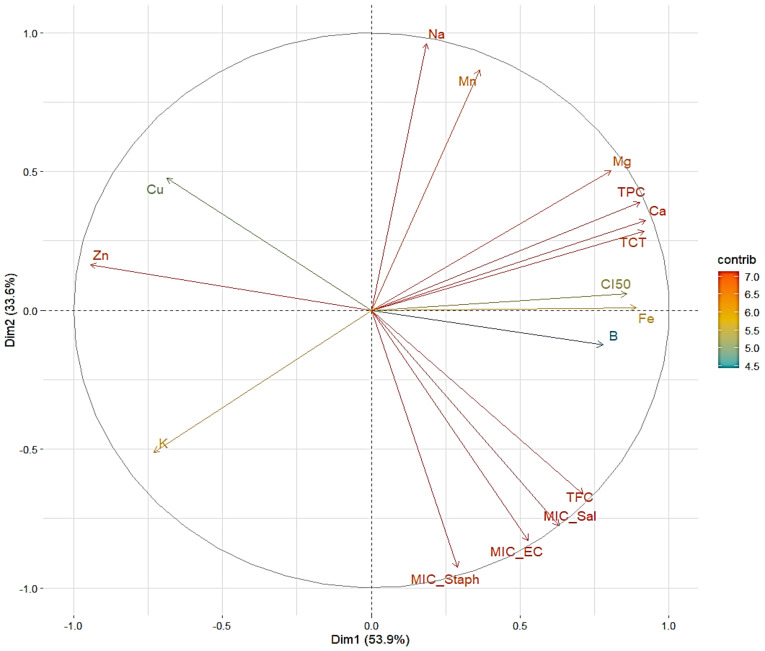
Principal component analysis describing the relationship between the studied parameters of *T. vulgaris*, *R. officinalis*, *P. anisum*, and *F. Phenolic* compound contents (TPC, TFC, and TTC), antioxidant activity, mineral elements, and antibacterial activity.

A plane defined by Dim1 (53.9%) and Dim2 (33.6%), accounting for 87.5% of the variation in Figure 3, is used to depict the distribution of variables in this PCA graphic. The arrows show how each variable contributes to the two main axes, with their color indicating the significance of their contribution (in terms of variance explained) based on the “contrib” scale; MIC_EC, MIC_Sal, MIC_Staph, and CI50 are key variables on Dim1 variables that exhibit a substantial positive contribution (on the right side of the graph), indicating a positive association among them. These factors appear to be related to studies of antibacterial and antioxidant activity against various bacteria. Their close positioning suggests similar variance and implies that they may work together to enhance the extract’s biological efficiency. On Dim1, however, K exhibits the reverse trend, indicating a negative correlation with the variables on the right. This suggests that higher potassium levels may be associated with reduced antioxidant or antibacterial activity. On Dim2, Na and Mn stand out due to their strong positive contributions. Their perspective suggests that, regardless of the biological activities as determined by IC_50_ or MIC, these variables may influence a different dimension. Additionally, a strong correlation was observed between the levels of tannins, total polyphenols, and minerals like Ca and Mg, as seen by the proximity of TPC, TCT, Mg, and Ca to the main axis of Dim1. This connection supports their potential contribution to the biological properties of the extracts under investigation. Furthermore, there is a weak association between biological activities and the positions of Fe and B along the Dim2 axis, although this relationship is not as strong as with other variables. Conversely, Zn and Cu in the left quadrant show a limited correlation with biological factors like IC_50_ or MIC. They appear to interact negatively with flavonoids and polyphenols, as their orientation is opposite to these variables. This could be due to the various inhibitory mechanisms or antagonistic effects. Overall, this PCA reveals three primary groupings: (1) a set of factors associated with biological activities (IC_50_, TFC, MIC); (2) a set of variables associated with important minerals (Ca, Mg, Mn, Na); and (3) variables with weak correlations (Zn and Cu).

## 3. Materials and Methods

### 3.1. Plant Material

For our study, we sourced all the plants from a local herbalist in Rabat, Morocco (33° 59′ 56.071″ N, 6° 50′ 57.055″ W) to ensure that all samples were completely organic. Each plant was meticulously air-dried for ten days in October and then stored in cardboard bags, each holding approximately 100 g of plant material. Before extraction, the plants were ground using a professional blender, and the resulting powder was passed through a 150 to 180-micron sieve to obtain a fine and homogeneous particle size. The prepared powder was then subjected to extraction using various solvents, including water, ethanol, and methanol. This rigorous process allowed us to obtain high-quality extracts essential for our analysis and research. Table 7 illustrates the classification and characteristics of different plants, focusing on their scientific and common names, botanical family, growth habits, cultivation status, and the parts of the plant used.

### 3.2. Preparation of Extracts

Each plant part was first ground into a fine powder to study the biochemical properties of the selected plants. Next, 10 g samples of each plant were extracted using 100 mL of an aqueous or organic solvent (ethanol and methanol). The extracts were obtained by maceration for 24 h. After maceration, the mixture was filtered using a Whatman filter paper (Hardened AHLESS, circle, 125 Ø, Clifton, NJ, USA) to separate the solid residues from the solution. The solvent was then evaporated using a rotary evaporator (model EVA180, IBX Instruments, Barcelona, Spain), and the extracts were stored at +4 °C until use. The extracts were then subjected to several types of analysis including the determination of total polyphenol content, evaluation of total flavonoid content, quantification of total tannin and catechins, antioxidant activity, and chromatographic analysis.

### 3.3. Determination of Total Polyphenol Content (TPC)

The total polyphenol content of the *T. vulgaris*, *R. officinalis*, *P. anisum*, and *F. vulgare* extracts was determined using the Folin–Ciocalteu (FC) reagent, following the methodology reported by [55]. A standard range of methanolic solutions was prepared from a gallic acid stock solution (0.5 g/L) with concentrations ranging from 0 to 200 μg/m. The plant extract (200 μL) was mixed with 1 mL of the FC reagent (10%), and the mixture was incubated in the dark for 20 min. Subsequently, 800 μL of Na_2_CO_3_ (7.5% (*w*/*v*)) was added along with a blank. The mixture was then stirred and incubated in the dark at a specified temperature for three hours. The absorbance was measured at 765 nm using a UV spectrophotometer (Peak instrument C-7200A, Shanghai, China). The results are expressed in μg gallic acid equivalent/mg of dry plant matter, based on the calibration curve of gallic acid.

### 3.4. Determination of Total Flavonoid Content (TFC)

The total flavonoid content in extracts of *T. vulgaris*, *R. officinalis*, *P. anisum*, and *F. vulgare* was determined as follows: 1.25 mL of distilled water, 0.075 mL of an aqueous solution of NaNO_2_ (5%, *w*/*v*), and 0.25 mL of extract solution were mixed. After 5 min, 0.15 mL of 10%, *w*/*v* AlCl_3_ solution was added, and 6 min later, 0.5 mL of 1 M NaOH was added to the mixture. The reaction mixture’s absorbance at 510 nm was measured against a blank after incubating for 30 min using a UV spectrophotometer (Peak instrument C-7200A) [56]. The results are expressed in μg of quercetin equivalent/mg of dry plant matter, based on the quercetin calibration curve.

### 3.5. Determination of Total Catechin Tannin (TCT)

The concentration of condensed tannins in extracts of *T. vulgaris*, *R. officinalis*, *P.anisum*, and *F. vulgare* was determined using the vanillin assay as described in the reference [55]. To obtain 50 μL of each extract, 1500 μL of a 4% vanillin/methanol solution was added and mixed. Next, 750 μL of concentrated HCl was added against a blank, and the mixture was allowed to react at room temperature for 20 min. The absorbance was measured at 500 nm by a UV spectrophotometer (Peak instrument C-7200A). The total concentration of condensed tannins was determined using a catechin calibration curve and expressed as micrograms of catechin equivalents per milligram of dry matter.

### 3.6. Antioxidant Activity

The antioxidant activity of the extracts was evaluated by their ability to scavenge DPPH (2,2-diphenyl-1-picryl-hydrazyl-hydrate) radicals [57]. To measure this, 0.5 mL of a 0.2 mM DPPH solution in ethanol was mixed with 2.5 mL of a diluted extract solution in ethanol, with a blank. Ascorbic acid was used as the reference substance. The mixture was stirred well and allowed to react for 30 min in the dark. After the reaction, the absorbance at 517 nm was measured. The DPPH radical scavenging activity was calculated using the following formula:

% Inhibition = [(Abs _Control_ − Abs _test_)/Abs _test_] × 100

### 3.7. Instrument and Chromatography Condition

A Thermo Fisher Vanquish LC system, composed of a binary pump, an autosampler, and a column oven C18 (150 × 2.1 mm 3 μm), was coupled with a Thermo Scientific Orbitrap Exploris 120 High-Resolution Mass Spectrometry (HRMS) (Waltham, MA, USA) The mobile phase was composed of solvent A (methanol with 0.1% formic acid) and solvent B (water with 0.1% formic acid). The gradient elution program was as follows: from 0 to 1.00 min, 70% A and 30% B; from 1.01 to 20.00 min, 100% B; from 20.01 to 25.00 min, 55% A and 45% B; and finally, from 25.01 to 40.00 min, 70% A and 30% B. The mobile phase flow rate was set to 0.30 mL/min, and the column temperature was maintained at 30 °C. A 3 µL injection volume was used for each analysis [58].

### 3.8. Minerals Determination

Minerals were determined by incinerating 10 g of the dried plant powder in a programmed muffle furnace, with temperatures gradually increasing from 100 °C to 450 °C for 7 h. After incineration, the material was cooled and treated with 3 mL of distilled water and then evaporated on a hot plate. The sample was then returned to the muffle furnace, starting at 200 °C and gradually increased to 450 °C over 2 h, during which 5 mL of hydrochloric acid was added. After evaporation on the hot plate, the resulting ash was dissolved in 10 mL of 0.1 mol/L nitric acid [59]. The mineral elements were then determined using a Varian AA240 GF-AAS (Palo Alto, CA, USA). A total of 9 elements were identified: potassium (K), calcium (Ca), magnesium (Mg), manganese (Mn), copper (Cu), iron (Fe), zinc (Zn), boron (B), and sodium (Na).

### 3.9. Antibacterial Activity

#### 3.9.1. Agar Diffusion Test

The antibacterial activity of hydroalcoholic extracts of *T. vulgaris*, *R. officinalis*, *P. anisum*, and *F. vulgare* was evaluated using the disk diffusion method. The bacterial strains *Escherichia coli* (ATCC25922), *Salmonella thyphimurium* (ATCC 14028), and *Staphylococcus aureus* (ATCC 25923) were inoculated into a Petri dish containing nutrient agar and incubated at 37 °C for 24 h. After bacterial growth, colonies were collected and diluted in sterile saline (0.9% NaCl) to a turbidity of 0.5 on the McFarland scale, equivalent to 1 × 10^8^ CFU/mL. Blank discs (6 mm in diameter) were impregnated with 10 µL of each hydroalcoholic extract and placed on pre-seeded Müller–Hinton agar plates. Positive and negative control antibiotic discs were also used to reveal the sensitivity of each bacteria, and the positive control included the following: Ciprofloxacin (Oxoid, 5 µg) for *E. coli* (ATCC 25922), Gentamicin (Oxoid, 10 µg) for *Salmonella* (ATCC 14028), and Oxacillin (Oxoid, 1 µg) for *Staphylococcus aureus* (ATCC 25923). The negative controls consisted of discs soaked in distilled water. The plates were incubated at 37 °C for 24 h [60].

#### 3.9.2. Determination of the Minimum Inhibitory Concentration (MIC) of the Extract

The plant extracts’ Minimum Inhibitory Concentration (MIC) was determined by preparing the extracts in microtubes and then dissolving the dried extract in distilled water. A 100 µL aliquot of culture medium (BHI) was added to each well of a 96-well microplate, followed by 100 µL of the test extract at a concentration of 10,000 µg/mL. Serial dilutions were performed in each well, resulting in the following concentrations: 500 µg/mL, 250 µg/mL, 125 µg/mL, 62.5 µg/mL, 31.25 µg/mL, 15.62 µg/mL, 7.81 µg/mL, 3.91 µg/mL, and 1.95 µg/mL. A bacterial suspension with a concentration of 10^8^ CFU/mL was prepared from a 24 h culture, and 10 µL suspension was inoculated into each well, including a positive control of Ciprofloxacin (Sigma-Aldrich, Merck, CAS: 93107-08-5 Darmstadt, Germany), Gentamicin sulfate salt (Sigma-Aldrich, Merck, CAS: 1405-41-0), Oxacillin sodium salt monohydrate (Sigma-Aldrich, Merck, CAS:7240-38-2 Darmstadt, Germany), and negative controls. The microplates were incubated at 37 °C for 24 h. After incubation, 10 µL of a 3-(4,5- dimethylthiazol-2-yl)-2, 5-diphenyltetrazolium bromide (MTT) solution (0.4 mg/mL in saline) was added, and the microplates were incubated further at 37 °C for 10 to 30 min [61].

### 3.10. Statistical Data Analysis

Statistical studies were conducted using RStudio software (version 2024.06.2+492). The parameters analyzed included TPC, TFC, TCT, and CI50, and the differences between these parameters were evaluated using an analysis of variance (ANOVA) test. According to the findings, every value was statistically significant (*p* < 0.05). In keeping with earlier research that showed ethanol extract’s efficacy as an extracting solvent for phenolic components and antioxidant activity, we exclusively employed it. To investigate the relationships between the different parameters measured and the ethanol extract samples, principal component analysis (PCA) was carried out. The FactoMineR and facto-extra packages in RStudio were used to process and visualize the data. It was possible to identify groups and commonalities among the various variables by projecting the samples onto a multidimensional space. The resulting biplots provided a clearer analysis of the correlations between the antioxidant activity and bioactive component levels.

## 4. Conclusions

This study demonstrated the abundance of phenolic compounds, flavonoids, and essential trace elements in *T. vulgaris*, *R. officinalis*, *P. anisum*, and *F. vulgare*, confirming their bioactive potential. The results revealed that ethanol was the most effective solvent for extracting these bioactive molecules, leading to significantly higher yields of TPC, TFC and TCT, which correlated with stronger antibacterial and antioxidant activities. These findings highlight the distinct chemical profiles and biological properties of each plant, emphasizing their potential for pharmaceutical, nutraceutical, and food applications.

A key perspective emerging from this study is the need to explore the synergistic effects of combining these plant extracts, as their bioactive components may interact to enhance their overall biological efficacy. Investigating these interactions between phenolic compounds, flavonoids, and trace elements could pave the way for the development of optimized antioxidant and antibacterial formulations, which could serve as natural alternatives to synthetic additives. Moreover, the incorporation of these plant-derived compounds into functional foods, dietary supplements, and therapeutic agents could enhance their potential health benefits.

Future research should focus on optimizing extraction techniques to maximize bioactive compound yield, studying the stability and bioavailability of these extracts, and assessing their potential toxicity through in vivo and clinical studies. Such investigations will be essential for validating their therapeutic applications and safety for human consumption. Additionally, advanced formulation techniques, such as nanoencapsulation, could be explored to improve the delivery and efficacy of these bioactive compounds.

Overall, these findings contribute to a growing body of evidence supporting the use of plant-based bioactive compounds in health-related industries. By advancing research on the formulation and application of these natural extracts, new opportunities may emerge for the development of eco-friendly and sustainable antimicrobial and antioxidant products, offering viable alternatives to synthetic chemicals in the pharmaceutical, food preservation, and cosmetic sectors.

## Figures and Tables

**Table 1:** Total polyphenols, flavonoids and catechin tannins content of the aqueous and organic extracts of *T. vulgaris*, *R. officinalis*, *P. anisum*, and *F. vulgare*.

Plant	Extract	TPC (μg GAE/mg E)	TFC (μg QE/mg E)	TCT (μg CE/mg E)
***T. vulgaris***	Aqueous	51.37 ± 0.04	126.45 ± 0.02	18.45 ± 0.4
	Ethanol	93.99 ± 0.05	111.37 ± 0.05	23.27 ± 0.1
	Methanol	70.96 ± 0.01	143.37 ± 0.02	20.38 ± 0.2
***R. officinalis***	Aqueous	58.68 ± 0.01	121.37 ± 0.04	5.62 ± 0.1
	Ethanol	75.79 ± 0.02	208.46 ± 0.16	21.17 ± 0.3
	Methanol	66.47 ± 0.02	99.61 ± 0.01	12.04 ± 0.1
***P. anisum***	Aqueous	37.70 ± 0.14	223.69 ± 0.02	4.01 ± 0.29
	Ethanol	67.71 ± 0.04	101.06 ± 0.05	16.85 ± 0.1
	Methanol	20.10 ± 0.11	25.50 ± 0.1	8.83 ± 0.01
***F. vulgare***	Aqueous	9.41 ± 0.10	17.71 ± 0.2	31.29 ± 0.03
	Ethanol	17.67 ± 0.02	170.8 ± 0.05	5.62 ± 0.01
	Methanol	14.22 ± 0.03	37.42 ± 0.1	10.43 ± 0.02

**Table 2:** Inhibitory concentration 50 (IC_50_) values for DPPH scavenging activity.

Plant	Extract	CI50 DPPH (μg/mL)
***T. vulgaris***	Aqueous	67.07 ± 1.07
	Ethanol	43.82 ± 0.51
	Methanol	49.41 ± 1.12
***R. officinalis***	Aqueous	15.87 ± 0.4
	Ethanol	12.79 ± 0.2
	Methanol	21.87 ± 0.4
***P. anisum***	Aqueous	72.33 ± 0.15
	Ethanol	84.71 ± 0.21
	Methanol	79.69 ± 0.22
***F. vulgare***	Aqueous	201.41 ± 0.10
	Ethanol	159.16 ± 0.2
	Methanol	165.23 ± 0.31

**Table 3:** The bioactive molecules detected in ethanol extracts of *T. vulgaris*, *R. officinalis*, *P. anisum*, and *F. vulgare*. D: Detected, ND: not detected.

Nom	Chemical Formulae	Structure	Retention Time	*T. vulgaris*	*R. officinalis*	*P. anisum*	*F. vulgare*	Bioactivity
**Tangeritin**	C_20_H_20_O_7_	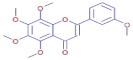	25.78	D	D	ND	D	Antimicrobial [25]
**Nobiletin**	C_21_H_22_O_8_	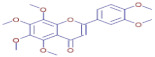	24.44	D	ND	ND	ND	Antibacterial [26]
**2-anisic acid**	C_8_H_8_O_3_	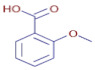	25.55	ND	ND	D	D	Antibacterial and antifungal [27]
**Thymol**	C_10_H_14_O	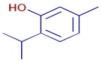	21.01	D	ND	ND	ND	Antioxidant and antibacterial [28]
**Ergosterol peroxide**	C_28_H_44_O_3_	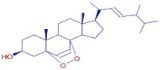	37.65	D	ND	ND	ND	antioxidant [29]
**D-(-)-quinic acid**	C_7_H_12_O_6_	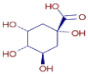	2.20	D	D	D	ND	Antioxidant [30]
**Naringenin**	C_15_H_12_O_5_	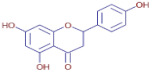	19.07	D	ND	ND	ND	Antimicrobial, antioxidant, and anti-inflammatory [31]
**Oleanolic acid**	C_30_H_48_O_3_	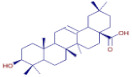	34.74	D	D	D	D	Antioxidant [32]
**Carvone**	C_10_H_14_O	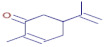	25.46	D	D	ND	ND	Antioxidant and antimicrobial [33]
**Glycitein**	C_16_H_12_O_5_	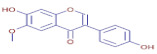	24.92	D	D	D	ND	Antimicrobial [34]
**Caffeic acid**	C_9_H_8_O_4_	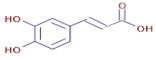	6.87	D	D	D	ND	Antibacterial and antioxidant [35]
**Apigenin**	C_15_H_10_O_5_	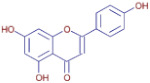	19.98	D	ND	ND	ND	Anticancer, Antioxidant, and antimicrobial [36]
**Maslinic acid**	C_30_H_48_O_4_	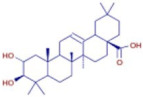	30.23	ND	D	ND	ND	Antibacterial [37]
**Ursolic acid**	C_30_H_48_O_3_	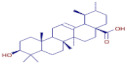	34.81	D	ND	ND	ND	Antioxidant [38]
**3,4-dihydroxyphenylacetic acid**	C_8_H_8_O_4_	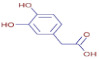	2.91	D	ND	ND	ND	Antioxidant [39]
**Salicylic acid**	C_7_H_6_O_3_	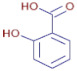	3.23	D	ND	D	D	Antibacterial [40]
**Rosmanol**	C_20_H_26_O_5_	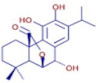	23.44	ND	D	ND	ND	Antioxidant and antimicrobial [41]
**Betulonic acid**	C_30_H_46_O_3_	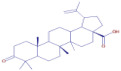	33.32	ND	D	D	D	Antioxidant [42]
**Eugenol**	C_10_H_12_O_2_	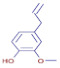	38.63	ND	ND	D	D	Antioxidant and antibacterial [43]
**(E)-p-coumaric acid**	C_9_H_8_O_3_	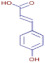	2.55	ND	ND	D	D	Antioxidant and antibacterial [44]
**Safrole**	C_10_H_10_O_2_	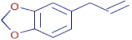	26.84	ND	ND	D	D	Antibacterial [45]
**Trans-anethole**	C_10_H_12_O	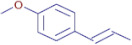	27.77	ND	ND	D	D	Antibacterial and antioxidant [46]
**Vanillin**	C_8_H_8_O_3_	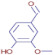	8.46	ND	ND	ND	D	Antibacterial [47]

**Table 4:** Mineral and metal contents in the investigated plants (mg/g dry matter).

	*T. vulgaris*	*R. officinalis*	*P. anisum*	*F. vulgare*
**Macroelement**				
**Ca**	20.823 ± 0.71	13.126 ± 0.52	12.528 ± 0.33	7.029 ± 0.24
**Mg**	3.354 ± 0.17	2.582 ± 0.11	2.796 ± 0.21	2.257 ± 0.15
**K**	0.658 ± 0.09	0.486 ± 0.06	0.752 ± 001	36.413 ± 0.98
**Na**	1.233 ± 0.13	0.700 ± 0.04	1.407 ± 0.091	0.728 ± 0.053
**Trace element**				
**Fe**	0.970± 0.11	0.32 ± 0.32	0.093 ± 0.004	0.088 ± 0.006
**Cu**	0.007 ± 0.002	0.003± 0.002	0.01± 0.01	0.010 ± 0.010
**Zn**	0.019 ± 0.03	0.017 ± 0.006	0.035 ± 0.004	0.041 ± 0.005
**Mn**	0.051 ± 0.01	0.02 ± 0.01	0.049 ± 0.005	0.021 ± 0.002
**B**	0.03 ± 0.01	0.01 ± 0.01	0.003 ± 0.002	0.009± 0.001

**Table 5:** Antimicrobial activity of the plant extracts against the tested bacterial strains.

		Zone of Inhibition (mm)		
Plant	Concentration (mg/mL)	*E. coli*	*Salmonella*	*Staphylococcus*
***T. vulgaris***	100	14.00 ± 0.50	7.00 ± 1.00	12.00 ± 0.50
	50	13.00 ± 0.0	-	7.50 ± 0.50
	25	9.00 ± 0.0	-	-
	12.5	7.00 ± 0.00	-	-
	6.25	-		-
***R. officinalls***	100	15.00 ± 1.50	12.50 ± 0.50	14.00 ± 1.00
	50	9.00 ± 0.05	11 ± 0.00	11.50 ± 0.50
	25	8.00 ± 0.00	Trace	10.00 ± 0.00
	12.5	7.00 ± 0.00	-	7.50 ± 0.00
	6.25	-	-	-
***P. anisum***	100	8.00 ± 0.00	7.50 ± 0.00	9.00 ± 0.50
	50	Trace	-	Trace
	25	-	-	-
	12.5	-	-	-
	6.25	-	-	-
***F. vulgare***	100	14.50 ± 1.50	12.50 ± 0.50	15.50 ± 1.00
	50	12.00 ± 0.50	11.00 ± 0.00	12.50 ± 1.00
	25	9.50 ± 0.50	7.00 ± 0.00	9.00 ± 0.50
	12.5	7.00 ± 0.00	Trace	7.00 ± 0.00
	6.25	Trace	-	-
**Water distilled**	100	-	-	-
	50	-	-	-
	25	-	-	-
	12.5	-	-	-
	6.25	-	-	-

**Table 6:** Minimum Inhibitory Concentration (MIC) of the plant extracts against selected bacterial strains.

Hydrolic Extract	MIC (µg/mL)		
	*E. coli*	*Salmonella*	*Staphylococcus*
***T. vulgaris***	7.81	31.25	15.62
***R. officinalls***	3.91	15.62	3.91
***P. anisum***	125.00	125.00	125.00
***F. vulgare***	31.25	62.5	3.91

**Table 7:** Description of the studied AMPs, their common names, scientific names, botanical families, growth habits, and used parts.

Common Name	Scientific Name	Abbreviation	Botanical Family	Growth Habit	Wild/Cultivated	Part Used
Thyme	*Thymus vulgaris*	*T. vulgaris*	Lamiaceae	Herbaceous	Cultivated	Leaves and stems
Rosemary	*Rosmarinus officinalis*	*R. officinalis*	Lamiaceae	Woody shrub	Cultivated	Leaves and stems
Anis	*Pimpinella anisum*	*P. anisum*	Apiaceae	Herbaceous	Cultivated	Seeds
Fennel	*Foeniculum vulgare*	*F. vulgare*	Apiaceae	Herbaceous	Cultivated	Seeds

## Data Availability

The diverse data generated and analyzed during this work are available from the corresponding author on request.
